# NF-E2-related factor 1 suppresses the expression of a spermine oxidase and the production of highly reactive acrolein

**DOI:** 10.1038/s41598-025-96388-7

**Published:** 2025-04-21

**Authors:** Tomoaki Hirakawa, Megumi Taniuchi, Yoko Iguchi, Sudarma Bogahawaththa, Kiko Yoshitake, Shanika Werellagama, Takeshi Uemura, Tadayuki Tsujita

**Affiliations:** 1 Laboratory of Biochemistry, Faculty of Agriculture, Saga University, Saga, Japan; 2 The United Graduate School of Agricultural Sciences, Kagoshima University, Kagoshima, Japan; 3 Faculty of Pharmacy and Pharmaceutical Sciences, Josai University, Saitama, Japan

**Keywords:** Spermine oxidase, Spermidine, NRF1, Acrolein, Polyamine, Polyamine imbalance, Biochemistry, Cell biology

## Abstract

Polyamines (putrescine, spermidine, and spermine) are among the most abundant intracellular small molecular metabolites, with concentrations at the mM level. The ratios of these three molecules remain constant under physiological conditions. Stress (i.e. polyamine overload, oxidative stress, aging, infection, etc.) triggers the catabolic conversion of spermine to spermidine, ultimately yielding acrolein and hydrogen peroxide. The potential of acrolein to induce DNA damage and protein denaturation is 1,000 times greater than that of reactive oxygen species. We have shown that these polyamine metabolic pathways also involve the nuclear factor erythroid-2-related factor 1 (NRF1) transcription factor. In our chemically-inducible, liver-specific *Nrf1*-knockout mice, the polyamine catabolic pathway dominated the anabolic pathway, producing free acrolein and accumulating acrolein-conjugated proteins in vivo. This metabolic feature implicates SMOX as an important causative enzyme. Chromatin immunoprecipitation and reporter assays confirmed that NRF1 directly suppressed *Smox* expression. This effect was also observed in vitro. Ectopic overexpression of SMOX increased the accumulation of free acrolein and acrolein-conjugated proteins. SMOX knockdown reversed the accumulation of free acrolein and acrolein-conjugated proteins. Our results show that NRF1 typically suppresses *Smox* expression when NRF1 is downregulated, SMOX is upregulated, and polyamine metabolic pathways are altered, producing low molecular weight polyamines and acrolein.

**Supplementary Information:**

The online version contains supplementary material available at 10.1038/s41598-025-96388-7.

## Introduction

Polyamines are the predominant small-molecule metabolites in the subcellular compartments, with concentrations in the mM range. The presence of polyamines is conserved from bacteria to mammals. Polyamines are essential for protein and nucleic acid synthesis and contribute to stabilizing nucleic acids^1–4^ and mitosis^5^. The most prevalent polyamines in vertebrates are Putrescine (Put), Spermidine (Spd), and Spermine (Spm). Ornithine decarboxylase (ODC) catalyzes the synthesis of Put, the polyamine with the lowest molecular weight. Subsequently, Spd and Spm are synthesized via the elongation of Put with sequentially transferred aminopropyl residues from *S*-adenosyl methionine^6^. Simultaneously, distinct metabolic enzymes degrade long-chain polyamines, Spd, and Spm. Through these metabolic pathways, the ratios of these principal polyamines are maintained. However, degradation of Spm to Spd by spermine oxidase (SMOX) generates 3-aminopropanal and hydrogen peroxide (H_2_O_2_)^7,8^. Acrolein, the simplest unsaturated aldehyde, is produced via a non-enzymatic reaction between H_2_O_2_ and 3-aminopropanal^7,8^.

The existence of acrolein has been disregarded for a long time because of its rapid reactivity with intracellular macromolecules, including genomic DNA^9^, proteins^7,10^, and lipids^11,12^. Acrolein is over 1,000 times more aggressive than reactive oxygen species (ROS), such as H_2_O_2_, and, therefore, regional increases in acrolein concentration significantly affect macromolecules^13^. Furthermore, acrolein-conjugated macromolecules lead to protein instability, mitochondrial dysfunction, and cell membrane disruption^14–17^. Therefore, suppressing acrolein production can contribute to the prevention of various diseases and the maintenance of normal cellular metabolism and cell division^18,19^. Notably, the total amount of polyamines gradually reduces with age, and this decline is now recognized as a risk factor for age-related diseases, such as neurodegenerative diseases^20,21^, and stroke^16,22^. Therefore, many studies have attempted to maintain total polyamine levels through polyamine-rich foods, such as nattō, oatmeal, and by transplanting gut microbiota^23–26^. However, pharmacological and molecular biological approaches to maintain total polyamine levels remain scarce.

NF-E2-p45-related factor 1 (NRF1), a type 2 membrane protein in the Cap’n'Collar basic region leucine zipper (CNC-bZIP) group of transcription factors, is typically anchored to the endoplasmic reticulum membrane^27^. The endoplasmic reticulum-associated protein degradation system (ERAD), also known as HRD1, is an E3 ubiquitin ligase that regulates the NRF1 protein level^28^. Proteasome inhibitors, such as MG132, stabilize the NRF1 protein and force its translocation to the nucleus^29^. After entering the nucleus, NRF1 binds to genomic loci containing the antioxidative response element (ARE) sequence^30,31^, transactivating genes that ameliorate unfolded protein stress and maintain thiol level equilibrium^31–33^.

In conditional *Nrf1*-knockout mice, depletion of NRF1 proteins in the liver, neuron, and brown adipocytes leads to osteoporosis^30^, fatty liver^33–35^, cancer^36^, diabetes^37^, and neurodegenerative diseases^38^. In vivo investigations have highlighted that NRF1 is critical for cellular homeostasis^39–41^, although the key molecule(s) responsible for these phenotypes remain elusive. NRF1 knockout in mice is lethal, especially in late-stage development, which poses a hindrance in studying the physiological functions of NRF1^42^. To overcome this and elucidate the physiological functions of NRF1, a previous study used the rat *CYP1A1* enhancer-driven *Cre*-*loxP* system to establish drug-induced and liver-specific NRF1-deficient mice^33^. This inducible liver-specific *Nrf1*-knockout mouse model developed by our group is one of the best models for elucidating the effect of NRF1 loss of function in the liver; therefore, we are using this system to identify the key molecules of cellular stress.

A previous study using inducible liver-specific *Nrf1*-knockout mice revealed a significant accumulation of glutathione, suggesting that NRF1 protects against excess oxidative stress^33^. However, hepatocyte ballooning and apoptosis were greatly exacerbated in NRF1-knockout mice. These phenotypes are similar to those associated with nonalcoholic fatty liver disease (NAFLD)^33–35^. Therefore, to elucidate the mechanism by which NAFLD is induced in *Nrf1*-knockout mice, we performed a comprehensive small-molecule metabolite variant analysis and found that increased fatty acid uptake is responsible for NAFLD^33^. Polyamine supplementation and acrolein suppression have anti-inflammatory effects^43,44^, prevent disease progression^45^, and promote longevity^23–25,46,47^. Therefore, controlling the polyamine metabolic pathway regulates endogenous stress and maintains cellular and organ homeostasis. In this study, we reanalyzed the data on small-molecule metabolites in drug-induced and liver-specific *Nrf1*-knockout mice and found variations in the polyamine metabolic pathway.

In this report, we discovered suppression of NRF1 protein and the subsequent upregulation of Smox, thus leading to the accumulation of shorter forms of polyamines, resulting in the production of the cellular stressors, acrolein in vivo and in vitro. This study provides valuable insights regarding possible ways to maintain total polyamine levels.

## Results

### Alteration of the polyamine content in NRF1-knockout mice

**Fig. 1:**
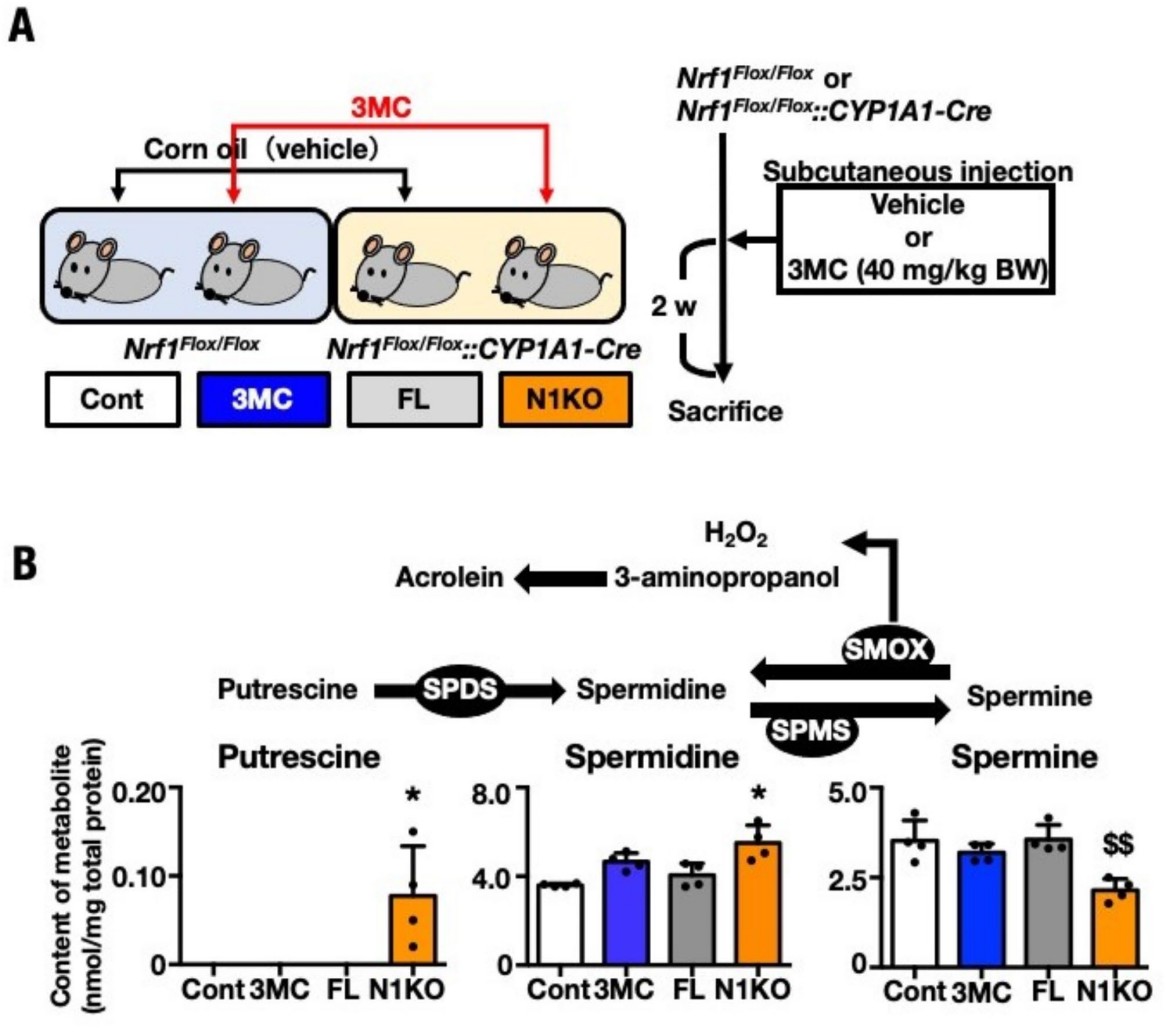
Increment of the short-length polyamine composition in NRF1-knockout mouse liver. (**A**) Schematic design of the process to obtain NRF1-knockout mouse liver. To ensure NRF1-knockout effect in *Nrf1*^*F/F*^*::1A1-Cre* + 3MC (N1KO) liver, three series of control groups, *Nrf1*^*F/F*^ + Vehicle (Cont), *Nrf1*^*F/F*^ + 3MC (3MC), and *Nrf1*^*F/F*^*::1A1-Cre* + Vehicle (FL), were used as control for overall, 3MC injection, and *1A1-Cre*-leaking effects, respectively. (**B**) Polyamine content in the livers from, Cont, 3MC, FL and N1KO. Liver metabolites were prepared with methanol. Extracts were subject HPLC using a polyamine column and the content of Put, Spd, and Spm were measured using respective standards. Each polyamine content is indicated as means ± SEM (nmol/mg protein) (n = 4). The statistical significance of results, compared with values from the Cont was calculated using one-way ANOVA with Dunnett’s test. ^*^, significant increase, *P* = 0.05 to 0.01; ^$^, significant decrease, *P* = 0.05 to 0.01, ^$$^, *P* = 0.01 to 0.001.

To examine hepatic polyamine levels during NRF1 depletion, we obtained the 3MC-inducible and liver-specific NRF1-knockout mice series [i.e., *Nrf1*^*F/F*^ + Vehicle (Cont), *Nrf1*^*F/F*^ + 3MC (3MC), *Nrf1*^*F/F*^*::1A1-Cre* + Vehicle (FL), *Nrf1F/F::1A1-Cre* + 3MC (N1KO)] as previously described in literature (Fig. 1A)^33^. The polyamine levels in these mice strains were determined using HPLC, which revealed an elevation in Put and Spd levels and a decrease in Spm in the livers of N1KO (Fig. 1B). Therefore, short-length polyamine composition increased in N1KO and the Spm-to-Spd degradation pathway was accelerated, leading to free acrolein induction via 3-aminopropanol by a non-enzymatic reaction^7,8^.

### Long-chain polyamine degradation through the SMOX pathway becomes dominant in N1KO

**Fig. 2:**
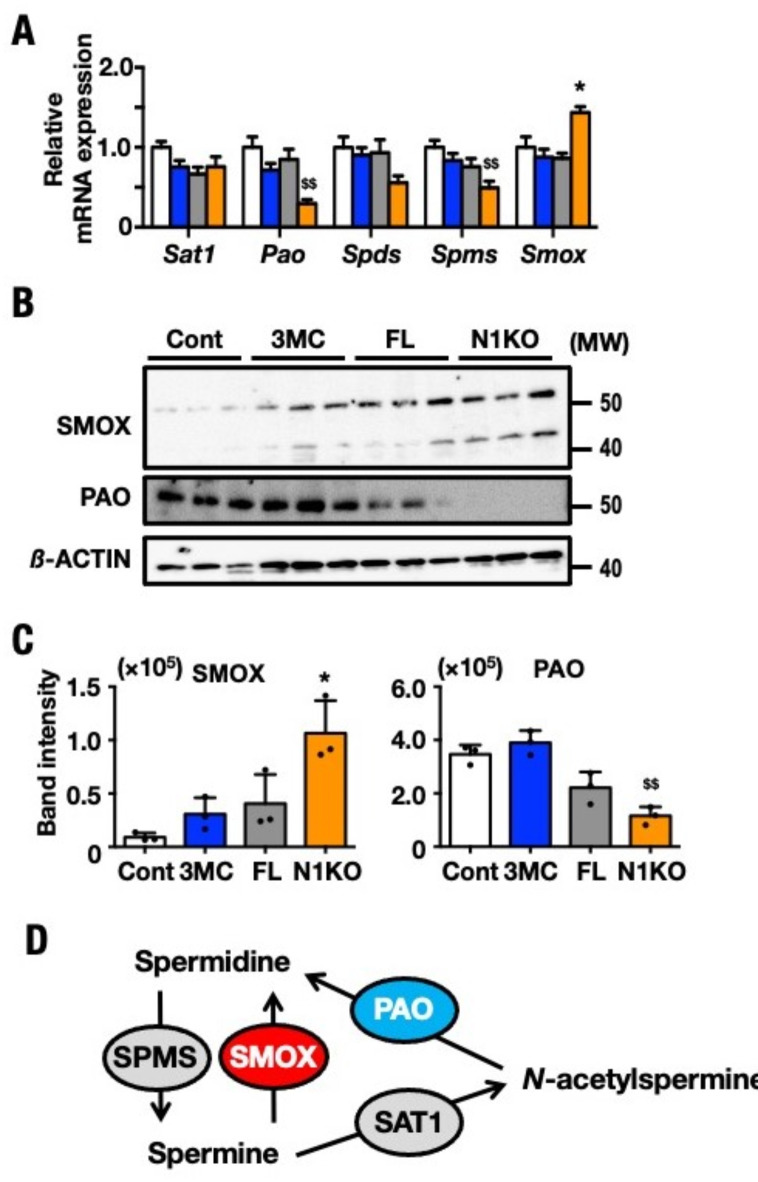
Increase of short-length polyamine content in N1KO was confirmed with mRNA expression profile and immunoblot. (**A**) mRNA levels of polyamine metabolism enzymes in Cont, 3MC, FL, and N1KO. These were determined by qRT-PCR. The results are normalized against mRNA level of *β-actin* and are shown mRNA expression level relative to that of Cont. (designated 1.0 for each gene). Histogram bar pattern employed for the mouse groups are indicated as white bars (Cont), blue bars (3MC), gray bars (FL), and orange bars (N1KO). Analyzed mice number of each four groups were 6. One of the three repetitions of the experiment is displayed, and results are expressed as means ± SEM. The statistical significance of results, compared with values of Cont was calculated using one-way ANOVA with Dunnett’s test. ^*^ and ^$^, *P* = 0.05 to 0.01; ^**^ and ^$$^, *P* = 0.01 to 0.001. (**B**) Immunoblot analyses of polyamine metabolic enzymes, SMOX and PAO. Protein molecular weights are shown on the right side of the blots. (**C**) The band signal intensity of SMOX and PAO was determined by ImageJ Fiji and normalized with that of β-ACTIN. Histogram bar patterns employed for the mouse groups were indicated as white bars (Cont), blue bars (3MC), gray bars (FL) and orange bars (N1KO). Protein molecular weights are shown on the right side of the blots. Data for each four groups were provided independent four mice. One of the triplicates of experiments is displayed and results are expressed as means ± SEM. The statistical significance of results compared with values of Cont was calculated using one-way ANOVA with Dunnett’s test. ^*^, *P* = 0.05 to 0.01. (**D**) Summary of the impact on polyamine metabolic pathways in N1KO. The content of individual polyamines and the protein level of metabolic enzymes are superimposed. The polyamines and protein levels in N1KO are compared with those in control mice (i.e., Cont, 3MC, or FL). Protein levels of polyamine-metabolizing enzymes (from **B**) are superimposed. Increased amounts are indicated in red, while reduced amounts are in blue, and no-change groups are gray in N1KO.

To determine the state of polyamine metabolism, we evaluated the mRNA expression and protein levels of polyamine metabolic enzymes in the livers from Cont, 3MC, FL, and N1KO using immunoblotting and RT-PCR. mRNA levels of *Spms* and *Pao* were significantly decreased in N1KO compared with other *Nrf1*-knockout mouse strains. In contrast, the *Smox* mRNA level was upregulated in N1KO (Fig. 2A). Using immunoblots, the levels of PAO and SMOX proteins were assessed simultaneously. SMOX protein level was significantly upregulated, whereas PAO protein levels were significantly downregulated in N1KO (Fig. 2B and C). RT-PCR and immunoblot results demonstrated that the Spm-to-Spd degradation was accelerated in N1KO, resulting in the production of free acrolein. Protein levels from Fig. 2B were superimposed on the polyamine pathways and the metabolic enzymes (Fig. 2D). The increase in the protein levels is denoted in red and the decrease in protein levels is denoted in blue. This experiment showed that the polyamine oxidation pathway becomes more prevalent in N1KO than the pathway via SAT1 and PAO. This SMOX-dominant degradation system increases the content of short-chain polyamines and the specific synthesis of free acrolein.

### Acute inducible loss of NRF1 in mouse liver causes accumulation of free acrolein and acrolein conjugated proteins

**Fig. 3:**
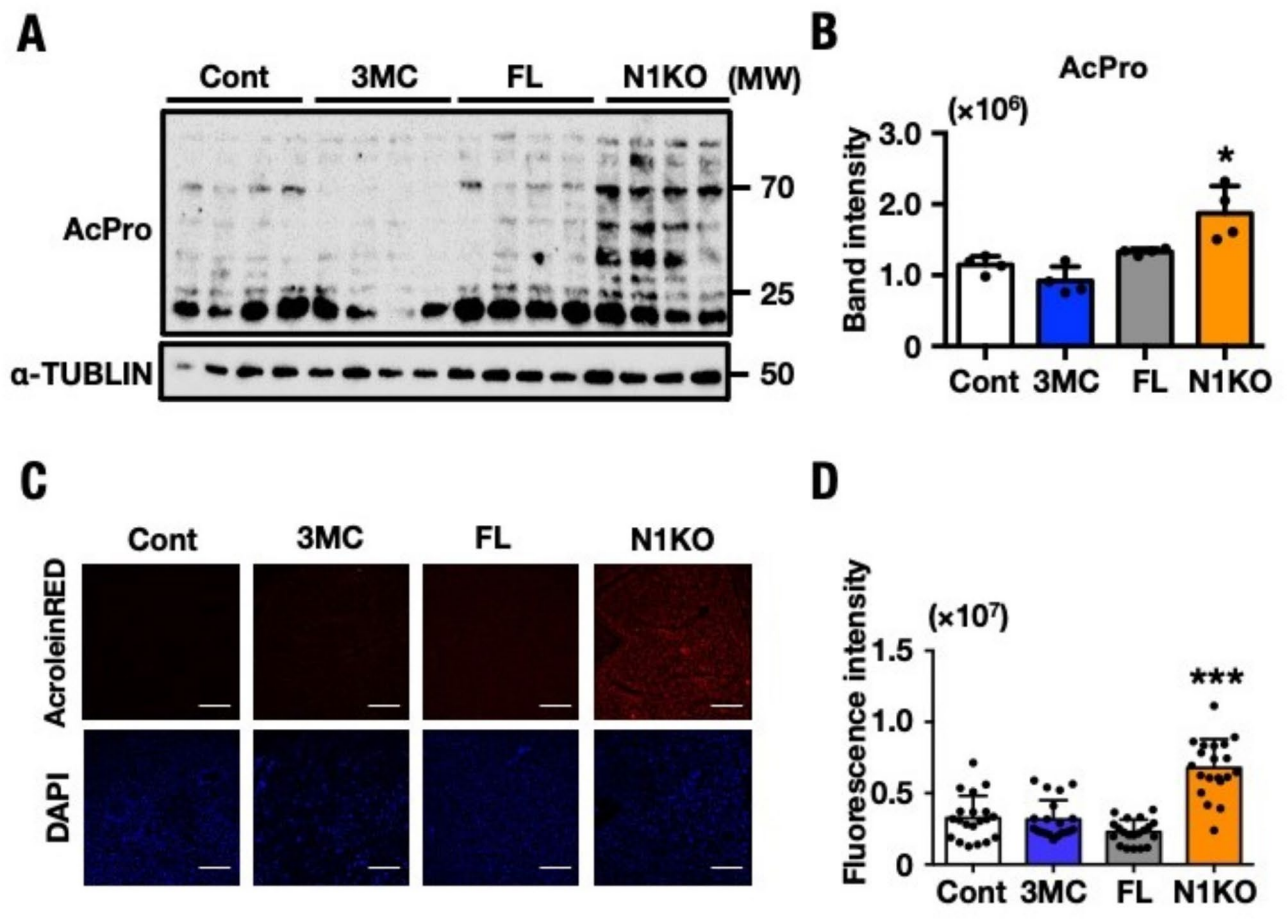
Accumulation of acrolein-conjugated proteins in N1KO. (**A**) Immunoblot analyses of acrolein-conjugated proteins. Protein molecular weights are shown on the right side of the blots. (**B**) The band signal intensity of acrolein-conjugated proteins were determined by ImageJ Fiji and normalized with that of α-tubulin. Histogram bar pattern employed for the mouse groups were indicated as white bars (Cont), blue bars (3MC), gray bars (FL) and orange bars (N1KO). One of the triplicates of experiments is displayed and results are expressed as means ± SEM (n = 4). The statistical significance of results, compared with values of Cont, was calculated using one-way ANOVA with Dunnett’s test. ^*^, *P* = 0.05 to 0.01. (**C**) Fluorescence staining was performed on sections from mice of all four groups using AcroleinRED and DAPI. Five images were taken from each individual mouse section using an inverted fluorescent microscope and processed through THUNDER Imaging Systems. *Red:* free acrolein, *Blue:* nucleus. Magnification: × 200. Scale bar = 132 µm. Data for each four groups were provided independent four mice. (**D**) Each dot represented as fluorescent intensity for individual image section. The values are indicated as means ± SEM. The statistical significance of results, compared with values of Cont, was calculated using one-way ANOVA with Dunnett’s test. ^***^, *P* = 0.01 to 0.001.

Based on the augment Spm to Spd current and the expression patterns of these metabolic enzymes, we hypothesized that an ectopic increase in SMOX protein level caused the increased synthesis of free acrolein in N1KO. Typically, free acrolein targeted macromolecules, such as proteins containing lysine, histidine, and cysteine residues^48–50^. Free acrolein targeted amino acids of surrounding proteins nonspecifically, altering their activity^14,51^. In this experiment, we used a specific antibody to recognize acrolein-modified lysine residues and visualized all acrolein-conjugated proteins (AcPro). Immunoblot analysis revealed a significant accumulation of AcPro in N1KO (Fig. 3A). We also quantified the band intensity of AcPro using ImageJ Fiji for comparisons with Control mice; the results showed that the concentration of AcPro was 1.8-fold higher in N1KO compared with other *Nrf1*-knockout mouse strains (Fig. 3B). These results validated the results of the analysis of polyamine levels using HPLC, gene expression alterations, and protein modifications in enzymes related to polyamine metabolic pathways. The activation of the long-chain polyamine degradation pathway not only produced free acrolein but also resulted in hydrogen peroxide accumulation as a byproduct. Further, free acrolein was significantly increased and the fluorescence intensity was two-fold higher in the livers of N1KO compared with that in the other three control mice strains (Fig. 3C and D). These results demonstrated that NRF1 downregulation leads to an increase in free acrolein accumulation and AcPro adducts in vivo.

### NRF1 regulates *Smox* transactivation directly

**Fig. 4:**
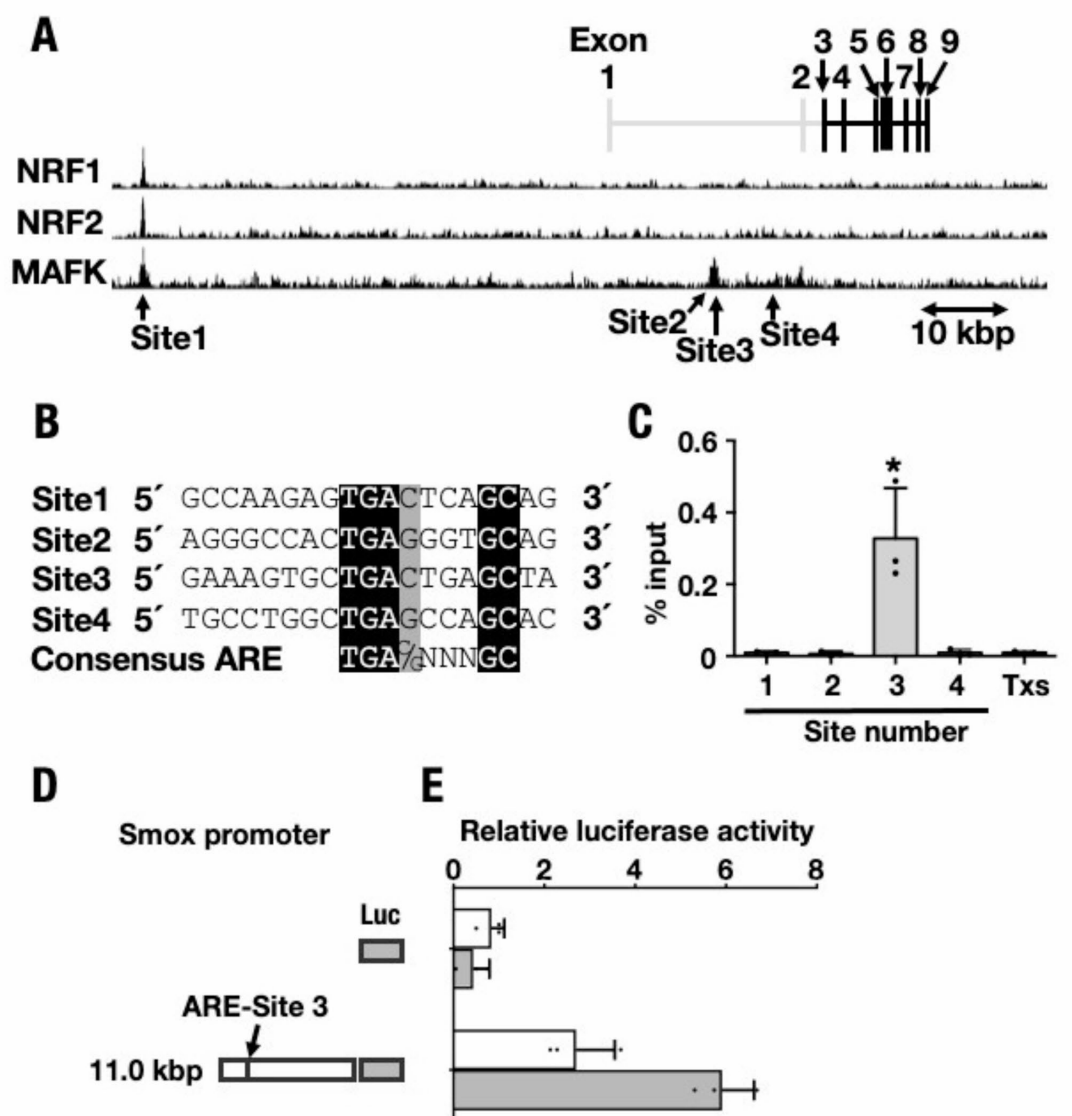
NRF1 directly binds to *Smox* genomic locus to suppress the expression. (**A**) Putative NRF1-binding sites around the *Smox* genomic region are predicted from the ChIP-seq data for MAFK, NRF1, and NRF2. ChIP-seq profiles of MAFK from Raw 264.7 cells and ES cells, NRF1 from MEF cells, NRF2 from Macrophages obtained from the Peak Browser of ChIP-Atlas, http://chip-atlas.org. Significant peaks that contain ARE consensus sequence are depicted as horizontal black bars and designed site 1, site 2, site 3, and site 4. *Smox* gene is constructed by seven parts of exon indicated with black and intron indicated with gray in Fig. 3A. Start codon in present in Exon 3. (**B**) The ARE sequences from predicted NRF1-binding sites indicated in panel A are aligned. Nucleotides that are conserved or similar between site 1, site 2, site 3, and site 4 are indicated as white letters on a black background or black letters on a gray background, respectively. (**C**) ChIP-qPCR experiment performed with an anti-NRF1 antibody. Specific primer sets were employed in qPCR from predicted DNA to detect site 1, site 2, site 3, site 4. *Txs*, genomic region in the third intron of *Txs* was used as a negative control. Analysis of each of the four groups of mice (n = 6). One of the triplicates of experiments is displayed, and results are expressed as means ± SEM. The statistical significance of results, compared with values of Cont, was calculated using one-way ANOVA with Dunnett’s test. ^*^, significant change, *P* = 0.05 to 0.01; ^**^, *P* = 0.01 to 0.001. (**D**) Schematic illustration of reporter constructs used in panels E. (**E**) Luciferase reporter gene assay to measure *Smox* transactivation in Hepa1c1c7 transfected shLacZ (open bars) or Hepa1c1c7 transfected shNrf1 (filled bars) cells. These cells were transfected with the SMOX reporter constructs depicted in panel E, and firefly luciferase activity was determined the after 48 h recovery. Data were standardized with *Renilla* luciferase activity.

To determine whether NRF1 regulates *Smox* gene transcription directly, we examined the binding of NRF1 to the *Smox* gene regulatory region using chromatin immunoprecipitation (ChIP) analysis and luciferase assay. First, we analyzed MafK ChIP-sequencing (ChIP-seq) data using the Peak Browser of ChIP-Atlas (GRCm38/mm10, http://chip-atlas.org) because no data on NRF1 binding to the *Smox* gene was available. Based on the information, we identified four putative NRF1-binding sites, which we designated as 1, 2, 3, and 4, located upstream of a start codon (Fig. 4A). Each candidate site was aligned using Box Shade to confirm the presence of consensus ARE motifs (Fig. 4B). We isolated DNA from mouse liver and conducted a ChIP experiment using an anti-NRF1 antibody and site-specific primer sets (Table S2). We also prepared primers specific for a negative site within *Txs* as a negative control. This ChIP experiment demonstrated that NRF1 was exclusively bound to site 3 upstream of exon 2, effectively suppressing *Smox* expression even under normal conditions. Other candidate sites 1, 2, and 4 and the negative control *Txs* lacked specific signals (Fig. 4C).

Second, we analyzed the activity of the SMOX promoter, which contains the NRF1 binding site 3, by ligation upstream of the firefly luciferase (Luc) (Fig. 4D). We prepared two reporter plasmids that contained starting ATG codon (− 10,936 to + 1) (SMOX-11-Luc) just upstream of SMOX. We transfected these reporter plasmids into Hepa1c1c7 cells with shNrf1 and measured Luc activity. The results showed that the Luc reporter gene expression from SMOX-11-Luc was significantly upregulated in Hepa1c1c7 transfected with shNrf1 plasmid, but a corresponding increase was not observed in the Hepa1c1c7 transfected control shRNA (shLacZ) plasmid (Fig. 4E). Therefore, we confirmed that NRF1 directly suppressed *Smox* expression under physiological conditions by binding to the 5’ regulatory site 3 ARE motif in *Smox*.

*Spms* and *Pao* genes contributed to Spm levels, and the expression of those genes was downregulated in N1KO (Fig. 2A). To determine whether NRF1 directly regulated *Spms* and *Pao* transactivation, we performed NRF1 ChIP analysis. Since no information on *Spms, Pao,* and NRF1 was available, we first analyzed MafK ChIP-seq data on the Peak Browser of ChIP-Atlas (GRCm38/mm10, http://chip-atlas.org). For *Spms,* four NRF1-binding candidate sites designated as sites 1, 2, 3, and 4 were identified (Fig. S1A). *Pao* had two candidate binding sites designated as sites 1 and 2 (Fig. S2A). All candidate sites were located in the upstream start codon (Figs. S1A and S2A). These candidate sites had well-conserved consensus ARE motifs (Figs. S1B and S2B). These ChIP experiments indicate that the expression of neither *Pao* nor *Spms* is controlled by NRF1 directly (Figs. S1C and S2C).

### *Nrf1* knockdown leads to SMOX protein increases and acrolein accumulation in vitro

We showed that the downregulation of NRF1 leads to acrolein accumulation along with *Smox* ectopic induction in a mouse model. To confirm the sequential molecular pathway for NRF1 downregulation, SMOX increases and accumulation of free acrolein, we knocked down *Nrf1* in Hepa1c1c7 cells using an shRNA expression system. The *Nrf1* shRNA tranfectants shown a significant increase in SMOX protein and free acrolein compared to the control *LacZ* shRNA transfectants (Fig. S3A–D). Therefore, these in vitro results support the hypothsis that the accumulation of free acrolein was associated with ectopic *Smox* upreguration through the downregulation of NRF1.

### *Smox* overexpression leads to acrolein accumulation in vitro

**Fig. 5:**
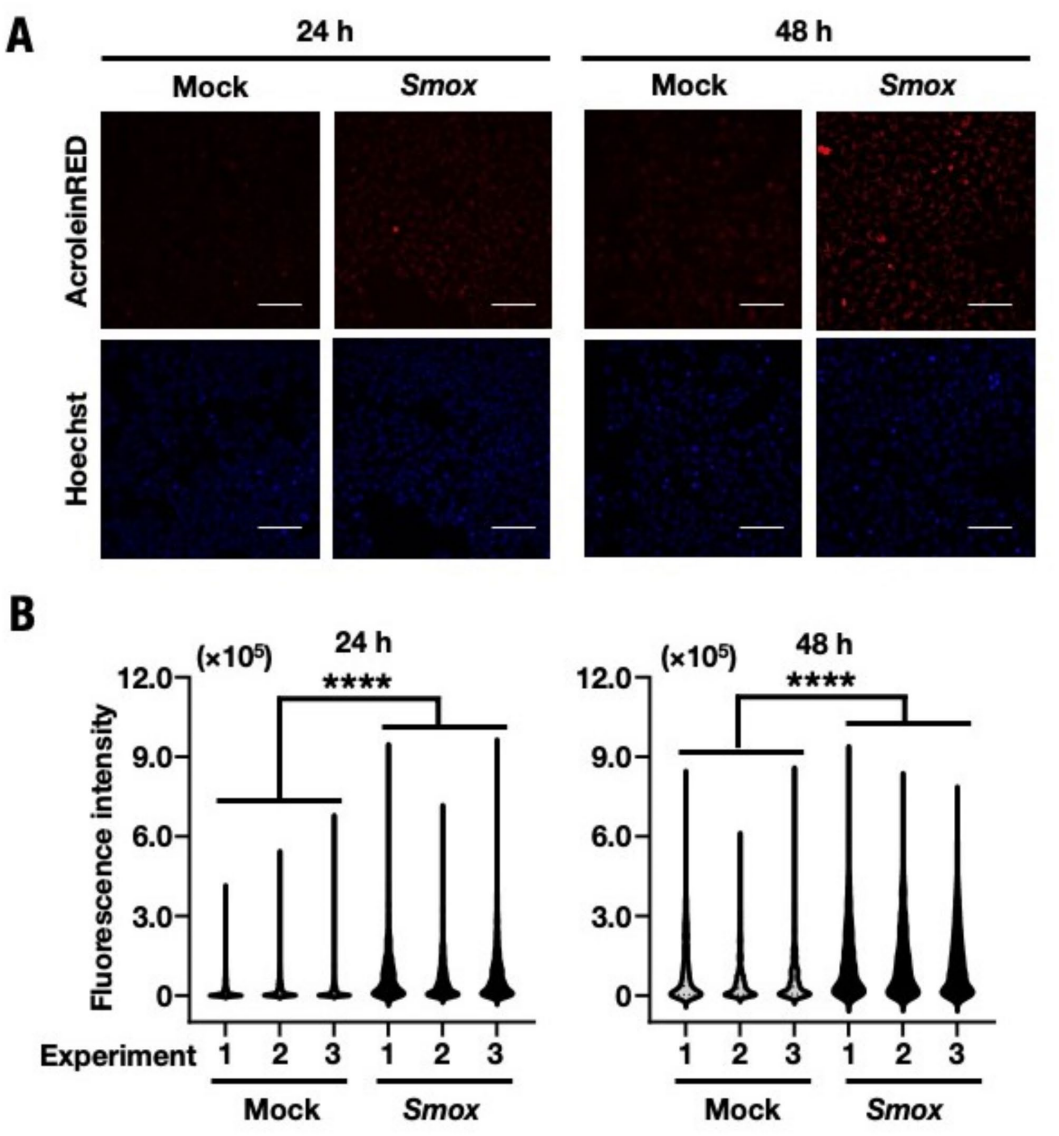
Increased free acrolein content in *Smox*-overexpressed HuH-7 cells. (**A**) Fluorescence image of overexpressed *Smox* or Mock transfectant HuH-7 was performed with AcroleinRED and Hoechst. Images were obtained by inverted fluorescent microscope and processed through THUNDER Imaging Systems after 24 and 48 h of transfection. *Red:* free acrolein, *Blue:* nucleus. Magnification: × 200. Scale bar = 132 µm. (**B**) Fluorescence intensities were quantified using ImageJ fiji. The content of acrolein in each individual cell was calculated using 1,000 cells in image fields. Each dot represents fluorescent intensity/cell. The values are indicated as means ± SEM. The statistical significance of results, compared with values from the Mock, was calculated using one-way ANOVA with Dunnett’s test. ^****^, *P* = 0.005 to 0.0001.

**Fig. 6:**
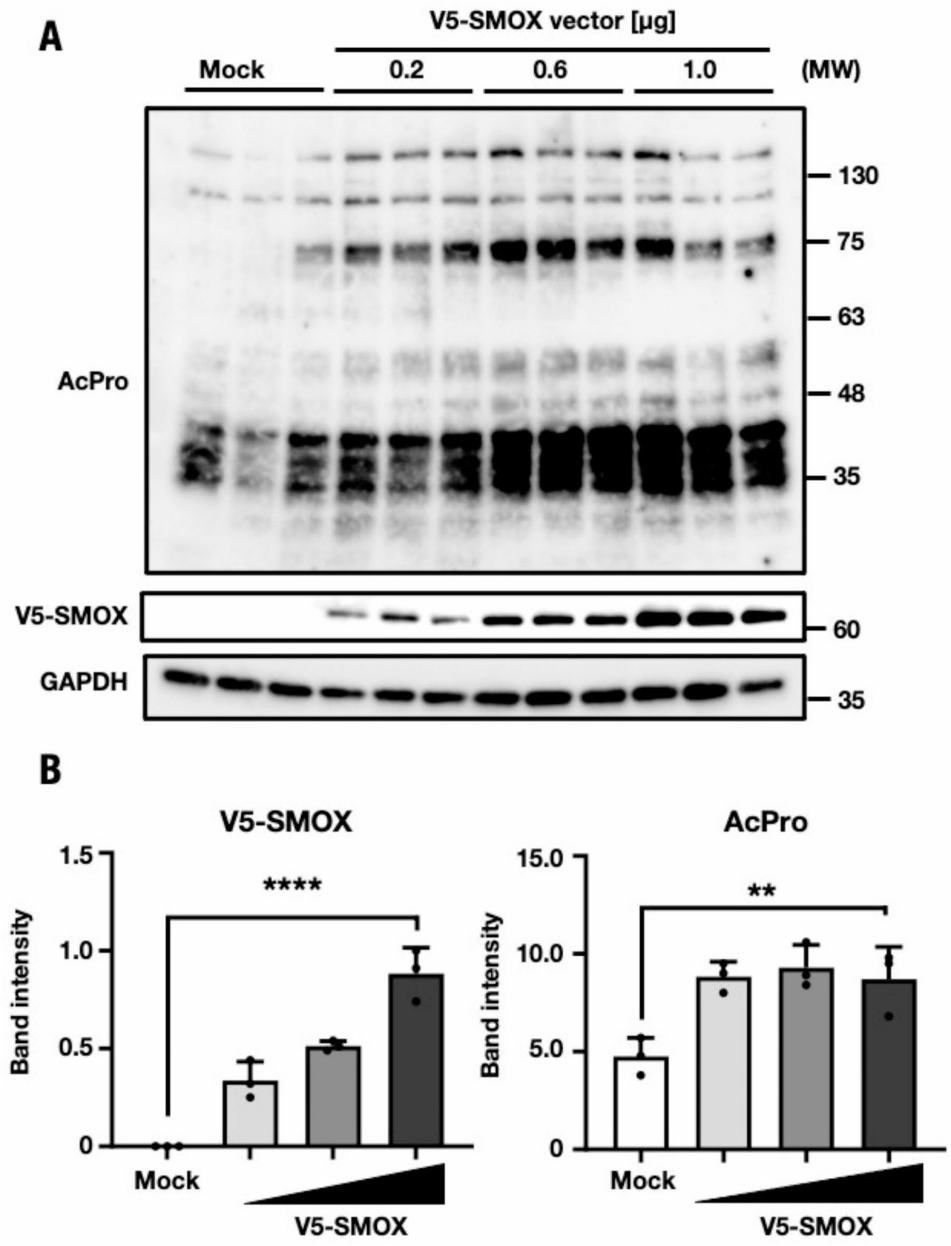
Increased the acrolein-conjugated protein content in SMOX-overexpressed HuH-7 cells. (**A**) Immunoblot analysis of antibody for acrolein-conjugated lysine and V5-tag. Aliquot of total lysates from three cells and equal amounts of lysates from the plasmid-transfected group were separated by SDS-PAGE (8% gel). The V5 tag indicated the level of SMOX overexpression. Equal loading was assessed by probing the blots with antibody against β-ACTIN which used as control in the experiment. Protein molecular weights are shown on the right side of the blots. (**B**) The band intensity of acrolein-conjugated protein and SMOX were determined using ImageJ Fiji, each band intensity were normalized with the band intensity of β-ACTIN. One of the triplicates (n = 3) of experiments is displayed and results are expressed as standard errors of the means ± SEM. The statistical significance of results, compared with values of Cont, was calculated using one-way ANOVA with Dunnett’s test. ^*^, *P* = 0.05 to 0.01.

In the N1KO mice liver, *Smox* overexpression was observed, leading to the accumulation of free acrolein and AcPro. To confirm this relationship between SMOX and the accumulation of free acrolein and AcPro, their levels were analyzed in *Smox*-overexpressed conditions. Free acrolein was significantly increased in the *Smox* overexpression vector transfectant (Fig. 5A and B). Also, immunoblot analysis revealed a dose-dependent accumulation of AcPro upon *Smox* expression (Fig. 6A and B). These experiments revealed that the accumulation of free acrolein and AcPro were directly related to *Smox* ectopic expression in the N1KO strain.

### *Smox* knockdown decreased acrolein production in vitro

**Fig. 7:**
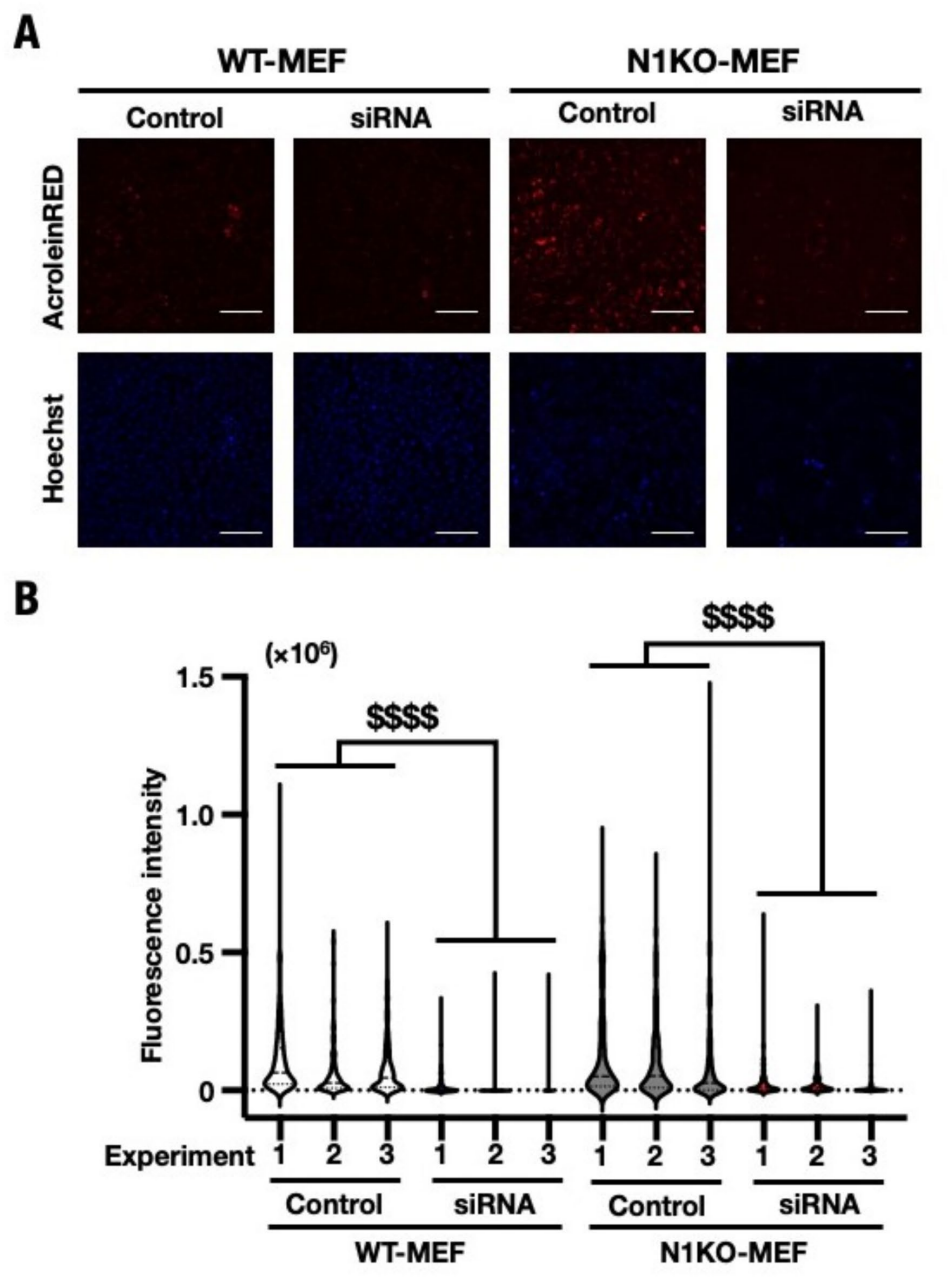
Decreasing the free acrolein content in *Smox* knockdown WT- and N1KO-MEF cells. (**A**) Fluorescence image of siRNA targeting *Smox* or control siRNA transfectant WT- and N1KO-MEF were performed with AcroleinRED and Hoechst. Images were obtained by inverted fluorescent microscope and processed through THUNDER Imaging Systems after 72 h of transfection. Bright field images were obtained phase contrast image, *Red* free acrolein, *Blue* nucleus. Magnification: × 200. Scale bar = 132 µm. *B*, Fluorescence intensities were quantified using ImageJ fiji. (**B**) The content of acrolein in each individual cell were calculated using 1000 cells in image fields. Each dot represented as fluorescent intensity/cell. The values are indicated as means ± SEM. The statistical significance of results, compared with values from the control was calculated using one-way ANOVA with Dunnett’s test. ^$$$$^, *P* = 0.005 to 0.0001.

**Fig. 8:**
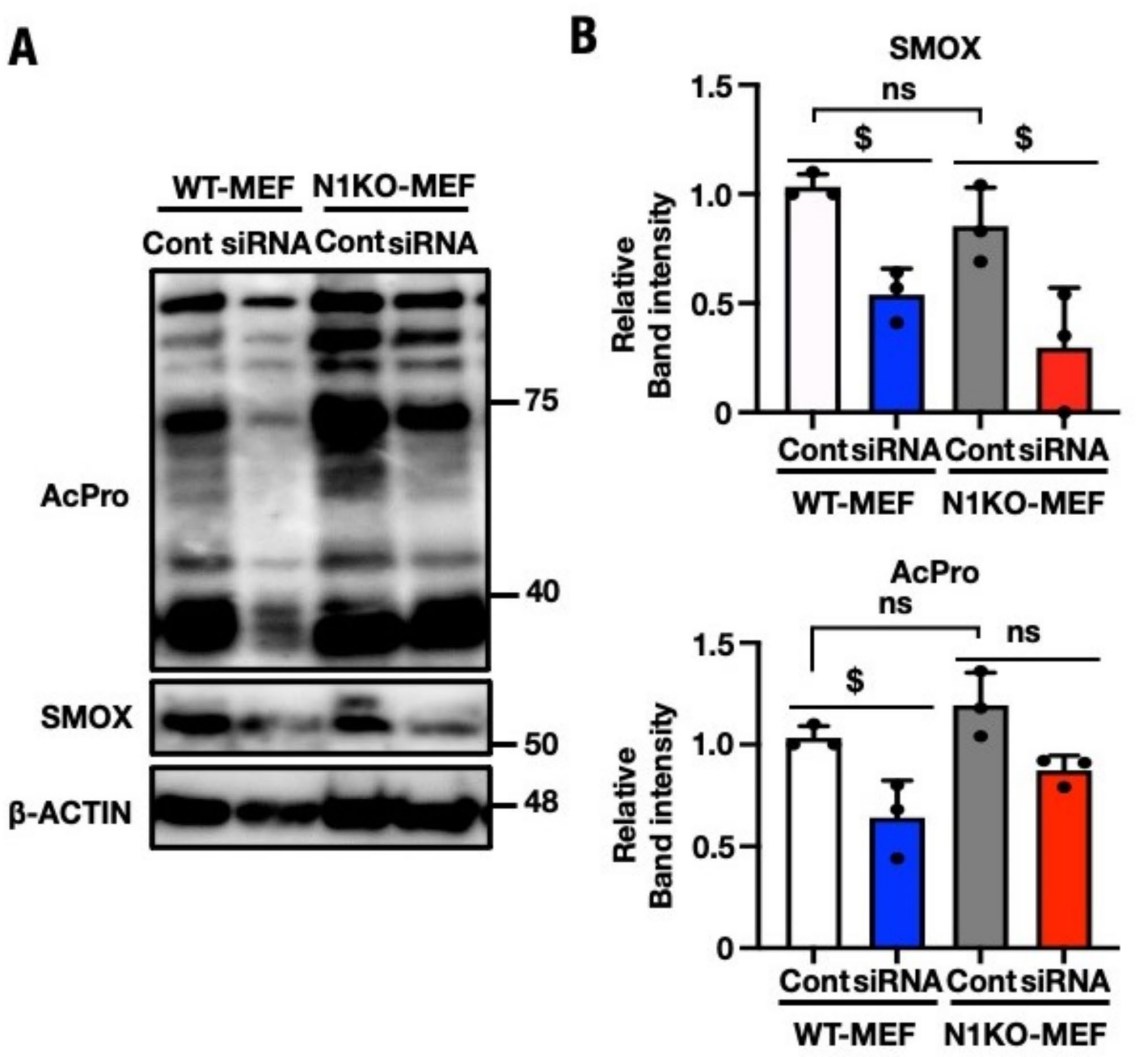
Decreasing the acrolein conjugated protein content in *Smox* knockdown MEF cells. (**A**) Immunoblot analysis of antibody for SMOX and Acrolein conjugated Lysine. Aliquot of total lysates from cells per equal amount of siRNA transected group were separated by SDS-PAGE (8% gel). Equal loading was assessed by probing the blots with antibody against β-ACTIN which used as control in the WT- and N1KO-MEF. Protein molecular weights are shewed right side of the blots. (**B**) The relative band intensity of acrolein conjugated protein and SMOX were determined by ImageJ Fiji, each band intensity were normalized with that of β-actin. One of the triplicates (n = 3) of experiments is displayed and results are expressed as standard errors of the means ± SEM. The statistical significance of results, compared with values from the control was calculated using one-way ANOVA.

To investigate the contribution of *Smox* to acrolein production*,* SMOX protein expression was studied in WT- and N1KO-MEF cells that were subject to knockdown siRNA transfection. SMOX protein levels were significantly reduced in WT- and N1KO-MEF cells that received *Smox* siRNA compared to those that received control siRNA (Fig. 8A). AcPro and free acrolein levels in *Smox* knockdown WT-MEF were also significantly downregulated when compared to the control transfectant (Figs. 7A and 8A). On the other hand, free acrolein levels in *Smox* knockdown N1KO-MEF were significantly downregulated when compared with the control transfectant (Fig. 7B); however, the AcPro level was not changed dramatically (Fig. 8B). These experiments showed that *Smox* expression levels are directly correlated with AcPro and free acrolein accumulation. In conclusion, NRF1 suppresses *Smox* expression under normal conditions to maintain the polyamine metabolic balance. Ectopic *Smox* overexpression such as *Nrf1*-knockout conditions, induces polyamine imbalance including the degradation of Spm to Spd and this metabolic change leads to the generation of intracellular free acrolein and a wide range of macromolecule modifications. Therefore, the investigation of *Smox* suppressor small molecules may be beneficial in the prevention of cellular damage due to intracellular free acrolein overproduction.

## Discussion

Under normal conditions, the ratios of Put, Spd, and Spm maintain the levels of polyamines, one of the major small molecule metabolites in vivo. This study confirmed that polyamine catabolism produces stressors, such as acrolein and H_2_O_2_, in the intracellular locus. These stress agents are frequently introduced exogenously to study specific stress responses in vitro, including oxidative stress^52,53^. Virus infections and genetic mutations are endogenous stress-elevation events that must be prioritized under physiological conditions when extrinsic stress pressure is low^53,54^. Therefore, suppressing and eliminating these toxic insults requires the elucidation of a sequential mechanism for stress generation in intracellular space. Among these regional intracellular stressors, singlet oxygen, generated by the mitochondrial electron transport chain, has been identified as a significant intracellular stressor^55,56^. Similar to singlet oxygen, acrolein has the potential to modify local macromolecules and is over a thousand times more toxic than H_2_O_2_^13^; however, its synthesis and detoxification pathways have not yet been studied. Endogenous acrolein directly modifies nucleic acids^9^ and the lysine, cysteine, and histidine residues on proteins^7,10,48,50^, inducing DNA damage^9^, ER stress^57^, inflammation^58^, and mitochondrial dysfunction^55,56^, all of which finally induce in vivo histological damage.

The polyamine degradation pathway includes various steps, such as Spm or *N*-acetyl-Spm to Spd and *N*-acetyl-Spd to Put to produce 3-aminopropanol or 3-acetoamidepropanol^58^. SMOX, SAT1, and PAO are the enzymes responsible for these processes, respectively. This catabolic process also produces H_2_O_2,_ which has been linked to various diseases^59,60^. Recently, acrolein has been identified as a risk factor for several diseases. Among the three enzymes mentioned above, the upregulation of SMOX and PAO has been established in several tissues during the aging process and validated by an increase in FDP-Lys, a bio-maker for acrolein^61^. Therefore, establishing a SMOX-suppression strategy is critical to prevent the accumulation of unfolded proteins and chronic inflammation in the aging process^17,62^.

Acrolein has been reported to make adducts with ACTIN, α, β-TUBULIN, and GAPDH, and these adducts can potentially disturb the functioning of these proteins^19,63^. A significant signal in our AcPro blots existed in the same molecular weight as ACTIN, α-TUBULIN, and GAPDH during SMOX overexpression. Therefore, we expect these modified proteins to induce cellular stress.

Long-term dietary consumption of Spd maintains polyamine levels, which increases longevity in yeast, flies, mice, and humans by enhancing mitochondrial respiratory performance^64^, neuron-inflammatory action^44^, wound healing function^65^, and autophagy mechanisms^46,66–69^. Recently, cell damage has been linked to an imbalance in polyamine levels toward degradation. From a pharmacological standpoint, a SMOX inhibitor, MDL72527, supplement has been reported to improve these characteristics^17^, and dieticians recommend taking food with higher polyamine contents, such as soybean and oatmeal^70,71^.

However, MDL72527 has been used as an inhibitor for not only SMOX but also PAO. The affinity of MDL72527 for PAO is 100-fold higher than for SMOX^72^. To develop a specific inhibitor for SMOX that does not inhibit PAO, establishes a proof of concept for treatment of diseases arising from intracellular accumulation of acrolein due to ectopic SMOX overexpression.

We confirmed increased conjugated and free acrolein accumulation in the NRF1*9*-knockout liver. Analysis of 3-Hydroxypropyl-mercuric acid (3-HPMA) levels has revealed that the primary systems for detoxifying free acrolein are glutathione and ALDH2 enzymes^73,74^. Glutathione (GSH), a tripeptide comprising glutamate, cysteine, and glycine, is the most abundant antioxidant. GSH captures free electrons from the electron transport chain machinery and forms oxidized glutathione dimers (GSSH) to detoxify ROS. GSH also contributes to acrolein detoxification by forming covalent bonds between the free thiol of GSH and the double bond of acrolein, forming nontoxic 3-HPMA^75,76^. Acrolein is also involved in Kelch-like ECH-associated protein 1 (KEAP1)–NRF2 system activation, where its toxicity is neutralized by anti-oxidative enzymes^51^. ALDH2, a key aldehyde-detoxification enzyme that converts acetaldehyde into acetic acid, contributes to acrolein detoxification^77^. Despite higher GSH content in NRF1-knockout mice liver^33^, free acrolein was assessed to be one of the most toxic agents for steatohepatitis. AcPro accumulation in NRF1 KO liver implied that GSH and ALDH2 did not completely detoxicate free acrolein. Free acrolein has been reported to rapidly modify protein functions before being detoxified by GSH and ALDH2 enzymatic reactions. Therefore, modifying the metabolic pathway before inducing aldehyde production is crucial for cellular defense.

Free acrolein production in WT- and N1KO-MEF cells was significantly reduced under *Smox*-knockdown conditions. Under the same conditions, AcPro levels were significantly downregulated in WT-MEF cells, but not in N1KO-MEF cells. In general, acrolein modification leads to impairment of the protein function and the modified proteins are degraded by the proteasome system. Studies both by our group and other groups have shown that NRF1 is essential for maintaining the protein levels of the proteasome subunit and its function^31,32^. Therefore, in this experiment, we conclude that even knocking down Smox levels in N1KO-MEF does not effectively remove AcPro.

## Conclusion

In conclusion, NRF1 typically inhibits SMOX expression. The downregulation of NRF1 counteracts the effects of SMOX suppression. Consequently, polyamine metabolism is redirected to generate low-molecular weight polyamines. This degradation pathway converts 3-aminopropanol to acrolein via a non-enzymatic reaction, which then attacks macromolecules, including proteins, DNA, and lipids. Therefore, regulating SMOX enzymes using NRF1 or other molecules will be advantageous to prevent tissue injury.

The novelty of this study lies in the observation that NRF1 suppresses SMOX expression and acrolein production by suppressing the tendency for polyamine degradation. To date, acrolein has been identified as a major exogenous stressor resulting from passive smoking and the presence of lipid hydroperoxides. Its extracellular recognition mechanism has also been studied. Further, the noteworthy aspect of this study is that it focused on the toxicity of acrolein produced in the intracellular space, rather than exogenous acrolein, and revealed that the polyamine degradation process is the main source of intracellular acrolein. Indeed, we showed that intracellular free acrolein rapidly forms an adduct with biological macromolecules, including proteins, and is extremely toxic. Associations between endogenous acrolein and diseases with poor prognosis, such as brain infarction, neurodegenerative diseases, and cancer, have also been reported, which supports these findings. Therefore, suppressing endogenous acrolein production is expected to prevent disease and ameliorate pathology. To further investigate this concept, a proof-of-concept study using a genetic model should be performed. Additionally, small molecules that can inhibit SMOX, which are yet to be used, could be applied to various therapies. Hereafter, it would be necessary to clarify these relationships and conduct verification experiments to improve the understanding of the pathology of these diseases.

## Materials and methods

### Mouse experiments and care

*Nrf1* conditional knockout mice (*Nrf1*^*Flox/Flox*^; RBRC10480) were obtained from the RIKEN Bioresource Research Center, Experimental Animal Division (Tsukuba, Ibaraki, Japan). *CYP1A1-Cre* mice (*CYP1A1-Cre*) were kindly provided by Professors Colin J. Henderson and C. Roland Wolf (Division of Systems Medicine, School of Medicine, University of Dundee, Dundee, UK)^76^. The AhR ligand 3-methylcholanthrene (3-MC) was used to induce *CYP1A1-Cre* as described previously^33^. 3-MC was dissolved in corn oil at a concentration of 4.0 mg/mL using a water bath ultrasonic sonicator and administered as a single subcutaneous (s.c.) injection to *Nrf1*^*Flox/Flox*^*::CYP1A1-Cre* mice at a dose of 40 mg/kg body weight. After 2 weeks, mice were sacrificed after being anesthetized with isoflurane and tissue samples were collected after reperfusion with normal saline. All mice were maintained under standard animal housing conditions with a 12 h:12 h light–dark cycle, with ad libitum access to water and an RM3 diet. All mouse experiments were approved by the Animal Care and Use Committee of Saga University under project number G2019-16-08. Our study was conducted and reported in accordance with ARRIVE guidelines, and all experiments were performed in accordance with relevant guidelines and regulations.

### Polyamine level measurement

Liver tissue samples (approximately 100 mg) were homogenized using a polytron homogenizer, and thereafter, subjected to a freeze–thaw cycle. The homogenate obtained was then suspended in 500 µL of 0.2 N perchloric acid and incubated for 30 min at 70 °C. The resulting suspension was centrifuged at 10,000 × g for 10 min at 4 °C. Thereafter, the supernatant collected was further centrifuged at 10,000 × g for 10 min at 4 °C. After the final supernatant was collected, 5 µL of 1 mM diaminohexane was added and polyamine concentrations were measured via HPLC using a TSKgel Polyaminepak column (Tosoh Bioscience, Tokyo, Japan) as previously described^46^. The polyamine levels were normalized by total protein concentration. The polyamine content was expressed as nmol/mg.

### RNA isolation and real-time qPCR

Total RNA was prepared from snap-frozen liver tissue samples using ISOGEN (Nippon Gene, Tokyo, Japan) according to the manufacturer’s instructions. Thereafter, a 1-µg aliquot of the total RNA was reverse transcribed using the Scriptase II 5 × Ready Mix system (Fast Gene, Tokyo, Japan), and for qRT-PCR using the KAPA SYBR FAST qPCR Master Mix system (Kapa Biosystems, Wilmington, MA, USA); cDNA was used as a template. The qPCR primers for *Sat1*, *Pao*, *Spds*, *Spms*, and *Smox* detection are listed in Table S1.

### Organelle fractionation and immunoblot analysis

Snap-frozen liver tissue samples (approximately 50 mg) were homogenized in ice-cold 1 × PBS (37 mmol/L NaCl, 8.1 mmol/L Na_2_HPO_4_, 2.68 mmol/L KCl, 1.47 mmol/L KH_2_PO_4_, pH 7.4) containing complete mini-EDTA-free protease inhibitor (Nacalai Tesque, Kyoto, Japan) via three to four strokes in a Dounce tissue grinder (Wheaton, Millville, NJ, USA). Thereafter, the liver homogenate was lysed by adding 2 × radioimmunoprecipitation assay (RIPA) buffer (50 mM Tris, 150 mM NaCl, 1% [v/v] NP-40, 0.5% [w/v] deoxycholic acid, 0.1% [w/v] SDS, pH 7.4), followed by three additional strokes. The debris was subsequently removed via centrifugation at 15,000 × *g* performed for 10 min at 4 °C. Thereafter, the protein content of the liver samples was determined using the bicinchoninic acid (BCA) protein assay reagent (Nacalai Tesque). The protein concentrations were adjusted to 1.0 mg/mL using RIPA buffer and 4 × sample buffer containing 200 mM dithiothreitol. In the next step, the samples were separated via SDS–polyacrylamide gel electrophoresis and transferred onto Immobilon membranes (FUJIFILM Wako, Osaka, Japan) for immunoblot analysis.

### Antibody

Immunoblot analysis was performed using the following antibodies: rabbit polyclonal anti-PAOX (PAO) (18972-1-AP; Proteintech, Rosemont, IL, USA), rabbit polyclonal anti-SMOX (15052-1-AP; Proteintech), mouse monoclonal anti-Acrolein antibody (5F6, JaICA, Tokyo, Japan), mouse monoclonal anti-α-TUBULIN (013-25033, FUJIFILM Wako), mouse monoclonal anti-β-ACTIN (010-27841; FUJIFILM Wako), mouse monoclonal anti-V5 antibody (#37–7500; Thermo Fisher Scientific, Waltham, MA, USA), Anti-mouse IgG, HRP-linked antibody (#7075; Cell Signaling Technology, Danvers, MA, USA), Anti-rabbit IgG, HRP-linked antibody (#7074; Cell Signaling Technology).

### Frozen section preparation and imaging

Snap-frozen liver samples were embedded into an optimal cutting temperature compound (Sakura fintech, Tokyo, Japan) and frozen at − 35 °C on an ice pack (Planet, Aichi, Japan) in liquid nitrogen. Thereafter, the samples were sectioned to 30-μm thick samples using CryoStar (Thermo Fisher Scientific) and mounted into a mass coat slide glass (Matsunami, Osaka, Japan). This was followed by staining using 20 mM AcroleinRED/DW (Funakoshi, Tokyo, Japan) and PureBlu DAPI Nuclear Staining Dye (BioRad, Hercules, CA, USA) for 30 min at 30 °C. Images were then obtained using a DMi8 inverted microscope (Leica Microsystems, Wetzlar, Germany) and processed using the THUNDER imaging system (Leica Microsystems).

### ChIP analysis

The ChIP assay was performed using mouse liver samples. The genomic DNA in the samples was fragmented indirectly using Cup Horn CH-063 (Tomy Seiko, Tokyo, Japan) and, thereafter, processed using the VCX Vibra-cell Ultrasonic Processor (Sonics & Materials, Newtown, CT, USA). Sonication was performed at the 70% level and at 4 °C for 10 cycles, with each cycle lasting 30 s. The debris obtained was subsequently removed via centrifugation at 1000 × *g* for 10 min at 4 °C. Further, immunoprecipitation was performed using the anti-NRF1 antibody. The sequence primer set for the thromboxane synthase gene (*Txs*) promoter was used as the negative control, and those flanking the ARE sequences in the promoters of *Smox* sites 1, 2, 3, and 4 are described in Table S2. The primers for *Pao* sites 1, 2, 3, and 4 and *Spms* sites 1 and 2 are described in Table S2. The amount of chromatin-associated DNA was estimated via qRT-PCR using the KAPA SYBR FAST qPCR Master Mix system (Kapa Biosystems).

### Reporter gene assay for *Smox* gene promoter

Hepa1c1c7 cells were plated in 24-well plates (4 × 10^4^ cells per well) and incubated for 16 h. They were then transiently transfected with SMOX luciferase reporter plasmids and pSUPER–LacZ or pSUPER–shNrf1 plasmid (1.0 µg) using Avalanche-Everyday Transfection Reagent (EZ biosystems, Baltimore, MD, USA) to decrease NRF1. To control for transfection efficiency, 10 ng of phRL-TK (Promega, Madison, WI, USA) was co-transfected with the reporter plasmids. Following a period of 48 h after transfection, *Smox* promoter activities were measured using the dual-luciferase reporter gene assay kit (Promega) using a single tube luminometer, Lumat LB 9507 (Berthold Technologies, Württemberg, Germany). Specific activity was calculated from light intensity measurements with a *Renilla* luciferase internal control.

### Cloning

pCMU SPORT6, which contains the mouse *Smox* sequence was obtained from Dharmacon (Lafayette, CO, USA). Specifically, the mouse *Smox* sequence was amplified using KAPA HiFi DNA polymerase (Kapa Biosciences) and inserted into pENTR/D-TOPO. Thereafter, it was swapped into pcDNA3.1-nV5-DEST (Thermo Fisher Scientific) using GatewayLR Clonase II enzyme. Thus, the final mouse expression vector was named pcDNA-nV5-Smox. Additionally, all the subcloned sequences were verified via Sanger sequencing using the Spectrum Compact CE System (Promega).

The mouse *Smox* genome upstream SMOX starting ATG codon (− 10,936 to + 1) (SMOX-11-Luc) was amplified using mouse genome and KOD FX (TOYOBO, Osaka, Japan). PCR product inserted into pGL3 basic vector (Promega) using In-Fusion Snap Assembly Master Mix (Takara Bio, Shiga, Japan). Thus, the reporter expression vector was named SMOX-11-Luc vector. Additionally, all the subcloned sequences were verified with Sanger sequencing using the Spectrum Compact CE System (Promega).

### Cell culture

The human hepatoma cell line, Hepa1c1c7, obtained from ATCC (Manassas, VA, USA), was cultured in MEMα medium (FUJIFILM Wako) containing 5% fetal bovine serum (Capricorn Scientific, Ebsdorfergrund, Germany), 100 unit/mL penicillin, and streptomycin (Nacalai Tesque) under humidified air containing 5% CO_2_ at 37 °C.

The human hepatoma cell line, HuH-7, obtained from ATCC (Manassas, VA, USA), was cultured in RPMI 1640 medium (FUJIFILM Wako) containing 5% fetal bovine serum (Capricorn Scientific), 100 unit/mL penicillin, and streptomycin (Nacalai Tesque) under humidified air containing 5% CO_2_ at 37 °C.

Mouse embryonic fibroblast (MEF) and *Nrf1*-knockout MEF cells were cultured in DMEM High glucose (FUJIFILM Wako) containing 5% Newborn Calf Serum (Capricorn Scientific), 100 unit/mL penicillin, and streptomycin (Nacalai Tesque) under humidified air containing 5% CO_2_ at 37 °C.

### Lipofection and sample preparation for imaging

Hepa1c1c7 cells were plated in 24-well plates (4 × 10^4^ cells per well) and incubated for 16 h. Thereafter, pSUPER–LacZ or pSUPER–shNrf1 plasmid (1.0 µg) using Avalanche-Everyday Transfection Reagent. This was followed by incubation for 6 h, after which 0.4 × 10^3^ transfectants were reseeded into a 96-well plate and incubated for 48 h.

In total, 1.8 × 10^5^ HuH-7 cells were plated onto 3-cm dishes and incubated for 16 h. Thereafter, 2.0 µg of pcDNA-nV5-*mmSmox* or pcDNA3.1 (empty vector) was introduced alongside Lipofectamine 3000. This was followed by incubation for 6 h, after which 0.4 × 10^3^ transfectants were reseeded into a 96-well plate and incubated for 48 h. Next, the cells were stained using 20 mM AcroleinRED (Funakoshi) and PureBlu Hoechst 33342 Nuclear Staining Dye (BioRad), and images were obtained using an inverted microscope and processed using the THUNDER imaging system (Leica Microsystems).

### RNA interference

Small interfering RNA (siRNA), which targets the mouse *Smox* was obtained from Thermo Fisher Scientific, and its sequence is shown in Supplementary Table S3. In total, 4.0 × 10^4^ WT-MEF or NRF1-knockout (N1KO)-MEF were plated into 24-well plates and incubated for 16 h. Thereafter, 2.75 pmol of siRNA targeting *Smox* or control siRNA was introduced alongside Avalanche-Omni Transfection Reagent (EZ Biosystems). After an incubation period of 48 h, transfectants were analyzed with fluorescent imaging and immunoblots.

### Statistical analyses

All data were analyzed using one-way analysis of variance (ANOVA) followed by the Dunnett multiple-comparison test using GraphPad Prism (GraphPad Software, La Jolla, CA, USA). Statistical significance of results was calculated using one-way ANOVA with Dunnett’s test when data for the two groups were unequally distributed, with *p* < 0.05 considered significant in all analyses.

## Electronic supplementary material

Below is the link to the electronic supplementary material.

Supplementary Material 1

## Data Availability

ChIP-sequence analysis (SRX3730280, SRX3937211, SRX2311119, DRX036575) are available in the ChIP-atlas database. Molecular structure of polyamines i.e., Put, Spd and Spm are avaible in the Worldwide Protein Data Bank (ID: PXD046396). cDNA and protein information can be found in NCBI website (Smox: NM_001177833.2, NP_001171304.1; Pao: NM_001346725.2, NP_001333654.1; Spms: NM_001359185.1, NP_001346114.1; Sat1: NM_001426002.1, NP_001412931.1; Spds: NM_009272.4, NP_033298.1; α-Tubulin: NM_011653.2, NP_035783.1; Nrf1: NM_001130450.1, NP_001123922.1; ß-actin: NM_007393.5, NP_031419.1; Other data are within the paper and its supplementary information files. The data generated during the study will be available from the corresponding author on reasonable request.

## Abbreviations

3MC: 3-Methylcholanthrene
AcPro: Acrolein-conjugated protein
ARE: Antioxidant response element
Aldh2: Aldehyde dehydrogenase 2
ATCC: American type culture collection
BCA: Bicinchoninic acid
CNC-bZip: Cap’n’Collar basic region leucine zipper
ERAD: Endoplasmic reticulum associated protein degradation system
FDP-Lys: Formyldehydropiperidino-lysine
H_2_O_2_: Reactive oxygen species
HPLC: High-performance liquid chromatography
3-HPMA: *N*-Acetyl-*S*-3-hydroxypropylcysteine
Keap1: Kelch-like ECH-associated protein 1
KO: Knockout
NAFLD: Non-alcoholic fatty liver disease
Nqo1: NAD(P)H dehydrogenase: quinone 1
NRF1: NF-E2 p45-related factor 1
NRF2: NF-E2 p45-related factor 2
Odc: Ornithine decarboxylase
Pao: Polyamine oxidase
PBS: Phosphate-buffered saline
Put: Putrescine
RIPA: Radioimmunoprecipitation assay
ROS: Reactive oxygen species
RT-qPCR: Quantitative reverse transcription-PCR
Sat1: Spermidine/spermine *N*^1^-acetyltransferase 1
SDS: Sodium dodecyl sulfate
Smox: Spermine oxidase
Spd: Spermidine
Spm: Spermine
Txs: Thromboxane synthase
