# Supplementary material for: NF-E2-related factor 1 suppresses the expression of a spermine oxidase and the production of highly reactive acrolein
Source: Sci Rep. 2025 Apr 21;15:12405. doi: 10.1038/s41598-025-96388-7 (PMC12012012; doi:10.1038/s41598-025-96388-7)
Supplement: Supplementary file 1 — Supplementary Material 1 [file 41598_2025_96388_MOESM1_ESM.docx]

**Table S1.** Primer sets for qRT-PCR experiments.

| **Gene** | **Primer** | **Sequence** |
| --- | --- | --- |
| *Sat1* | Forward primer | 5´–GAGAACACCCCTTCTACCACT–3´ |
|  | Reverse primer | 5´–GCCTCTGTAATCACTCATCACGA–3´ |
| *Pao* | Forward primer | 5´–TGGGCTGGATTGCATCTTGG–3´ |
|  | Reverse primer | 5´–AAAAGCGACCGTATCCTTGGG–3´ |
| *Spds* | Forward primer | 5´–CTTCCCCGTGGTGGACTAC–3´ |
|  | Reverse primer | 5´–TGCTCGGGTTTTTGCTACACA–3´ |
| *Spms* | Forward primer | 5´–GACCTTCAGAGTTACGACAGTG–3´ |
|  | Reverse primer | 5´–GAACTATGGGTGGTAATCGCTTC–3´ |
| *Smox* | Forward primer | 5´–GACAGTGCGGATGACCCTC–3´ |
|  | Reverse primer | 5´–GATAGATAGGATTCCCGTGGGAT–3´ |
| $\beta$*-Actin* | Forward primer | 5´–CGCGAGCACAGCTTCTTTG–3´ |
|  | Reverse primer | 5´–CATGCCGGAGCCGTTGTC–3´ |

**Table S2.** Primer sets for ChIP experiments.

| **Gene or Site** | **Primer** | **Sequence** |
| --- | --- | --- |
| *Smox* site1 | Forward primer | 5´–GCTTAGCCGGTCTCTGCTTC–3´ |
|  | Reverse primer | 5´–GGAAGTCCCTGAAATTTACCACATTAG–3´ |
| *Smox* site 2 | Forward primer | 5´–GAGGCTAAGCTTGCTGGAGAC–3´ |
|  | Reverse primer | 5´–CCACTTGCAAAAAATGAGGCTGTG–3´ |
| *Smox* site 3 | Forward primer | 5´–GCTGAGAGGCTGAGTTGTGTTC–3´ |
|  | Reverse primer | 5´–GAGGATACTGAAAGATGGATACCTAGAAC–3´ |
| *Smox* site 4 | Forward primer | 5´–TGGGCTGGATTGCATCTTGG–3´ |
|  | Reverse primer | 5´–AAAAGCGACCGTATCCTTGGG–3´ |
| *Pao* site 1 | Forward primer | 5´–GTTTAGGGACCACCCAGAGGTTATG–3´ |
|  | Reverse primer | 5´–GATTCTGTGGGCGGAACACATC–3´ |
| *Pao* site 2 | Forward primer | 5´–CGATGTGTTCCGCCCACAGAATC–3´ |
|  | Reverse primer | 5´–GTGATGCAATTAACAGACTACTCCAAG–3´ |
| *Pao* site 3 | Forward primer | 5´–GACATCAGTGAGAGTAGAAGAGAGG–3´ |
|  | Reverse primer | 5´–GGCAAGCATGACTTCAATTGCTATCTG–3´ |
| *Pao* site 4 | Forward primer | 5´–GAAGCTGATCGCCTCGTTAGTC–3´ |
|  | Reverse primer | 5´–GGGCATCCATGATCAGACCAAG–3´ |
| *Spms* site1 | Forward primer | 5´–GTATCTGCAGCTCACATTAGTCTGATG–3´ |
|  | Reverse primer | 5´–CTGATTTTGTTGGCCCTGGCTG–3´ |
| *Spms* site2 | Forward primer | 5´–CAGCCAGGGCCAACAAAATCAG–3´ |
|  | Reverse primer | 5´–CACAGGTACACCAGTCAGTCTC–3´ |
| *Txs* | Forward primer | 5´–CTTCCCCGTGGTGGACTAC–3´ |
|  | Reverse primer | 5´–TGCTCGGGTTTTTGCTACACA–3´ |

**Table S3.** siRNA for knockdown experiments.

| **Gene** | **Sequence** |
| --- | --- |
| *Smox* | 5´–CGGGAAUCCUAUCUAUCAAtt–3´ |

**Fig. 1S.** *Pao* was not suppressed by NRF1 directly during physiological condition. (**A**) Putative NRF1 binding sites around the *Pao* genomic region are predicted from the ChIP-seq data for MAFK, NRF1, and NRF2. ChIP-seq profiles of MAFK from Raw 264.7 cells and, NRF1 from MEF cells, and NRF2 from C2C12 cells obtained from the Peak Browser of ChIP-Atlas, http://chip-atlas.org. Significant peaks that contain ARE consensus sequence are depicted as horizontal black bars and designed Pao site 1, site 2, site 3, and site 4. *Pao* gene is constructed by ten parts of exon indicated with black and intron indicated with gray. Start codon contain in Exon 1. (**B**) The ARE sequences from predicted Nrf1 binding sites indicated in panel (**A**) are aligned. Nucleotides that are conserved or similar between site 1, site 2, site 3, and site 4 are indicated as white letters on a black background or black letters on a gray background, respectively. (**C**) ChIP-qPCR experiment performed with an NRF1/TCF11 antibody. Specific primers sets were employs in qPCR from predicted DNA to detect site 1, site 2, site 3, and site 4. *Txs*, genomic region in the third intron of *Txs*, was used as a negative control. Analysis of wild type of mice (n=3). One of the triplicates of experiments is displayed, and results are expressed as means ± SEM. The statistical significance of results, compared with values were calculated using one-way ANOVA with Dunnett’s test.

**Fig. 2S.** *Spms* was not suppressed by NRF1 directly during physiological condition. (**A**) Putative NRF1 binding sites around the *Spms* genomic region are predicted from the ChIP-seq data for MAFK, NRF1, and NRF2. ChIP-seq profiles of MAFK from Raw 264.7 cells and Trophoblast stem cells, NRF1 from MEF cells, NRF2 from C2C12 cells obtained from the Peak Browser of ChIP-Atlas, http://chip-atlas.org. Significant peaks that contain ARE consensus sequence are depicted as horizontal black bars and designed site 1, and site 2. Spms gene is constructed by twelve parts of exon indicated with black and intron indicated with gray. Start codon contain in Exon 1. (**B**) The ARE sequences from predicted NRF1 binding sites indicated in panel (**A**) are aligned. Nucleotides that are conserved or similar between site 1 and site 2 are indicated as white letters on a black background or black letters on a gray background, respectively. (**C**) ChIP-qPCR experiment performed with an NRF1/TCF11 antibody. Specific primers sets were employs in qPCR from predicted DNA to detect site 1, and site 2. *Txs*, genomic region in the third intron *Txs*, was used as a negative control. Analysis of wild type mice (n=3). One of the triplicates of experiments is displayed, and results are expressed as means ± SEM. The statistical significance of results, compared with values were calculated using one-way ANOVA with Dunnett’s test.

**Fig. S3.** Free acrolein increase in *Nrf1* knockdown condition *in vitro*.

(**A**) Immunoblot analyses of polyamine metabolic enzymes, SMOX. Protein molecular weights are shown on the right side of the blots. **(B)** The band signal intensity of SMOX was determined by ImageJ fiji and normalized with that of GAPDH. **(C)** Fluorescence image of shRNA targeting *Nrf1* or control shRNA transfectant Hepa1c1c7 were performed with AcroleinRED and Hoechst. Images were obtained by inverted fluorescent microscope and processed through THUNDER Imaging Systems after 48 h of transfection. Bright field images were obtained phase contrast image, *Red* free acrolein, *Blue* nucleus. Magnification: ×200. Scale bar = 132 µm. *B*, Fluorescence intensities were quantified using ImageJ fiji. **(D)** Fluorescence intensities were quantified using ImageJ fiji. The content of acrolein in each individual cell was calculated using 1,000 cells in image fields. Each dot represents fluorescent intensity/cell. The values are indicated as means ± SEM. The statistical significance of results, compared with values from the control shRNA, was calculated using one-way ANOVA with Dunnett’s test. ^****^, *P* = 0.005 to 0.0001.

**Fig. S4.** Uncropped immunoblot images.

Uncropped images of all immunoblots used in Figures 2B, 3A, 6A, 8A, and 3S are shown, along with the approximate extent of the cropped region.
